# The Effects of CrossFit^®^ Practice on Physical Fitness and Overall Quality of Life

**DOI:** 10.3390/ijerph22010019

**Published:** 2024-12-28

**Authors:** Manoel Rios, David B. Pyne, Ricardo J. Fernandes

**Affiliations:** 1 Superior School of Sport and Education, Jean Piaget Polytechnic Institute of the North, 4405-678 Vila Nova de Gaia, Portugal; 2 Centre of Research, Education, Innovation and Intervention in Sport and Porto Biomechanics Laboratory, Faculty of Sport, University of Porto, 4200-450 Porto, Portugal; ricfer@fade.up.pt; 3 Research Institute for Sport & Exercise, University of Canberra, Canberra 2617, Australia; david.pyne@canberra.edu.au

**Keywords:** CrossFit^®^, health, physical fitness, exercise, benchmark, workouts

## Abstract

We have examined the impact of CrossFit^®^ workout sessions on physical fitness, comparing the obtained outcomes with the recommendations of the American College of Sports Medicine. In addition, we provide suggestions to improve training monitoring, as well as practical applications for researchers, coaches and practitioners. CrossFit^®^ imposes high cardiorespiratory and metabolic demands, promoting improvements in circulatory capacity, oxidative metabolism and muscular endurance. Sustained elevations in heart rate contribute to cardiovascular conditioning, while a post-exercise hypotensive effect may help to reduce cardiovascular risks. Structured CrossFit^®^ programs have led to improvements in maximal strength and muscular endurance, with substantial increases in squat performance observed in both untrained and recreationally active individuals. In addition, CrossFit^®^ improves mental health through its motivating community. However, the high metabolic demands, increased creatine kinase levels and reduced performance in the countermovement jump reveal that muscle damage and neuromuscular fatigue can persist for up to 48 h. Balancing these intense sessions with adequate recovery is crucial, as improper management may lead to overtraining and compromise fitness gains. Future research should explore long-term cardiovascular adaptations, differences in gains and recovery between males and females and the application of real-time biomarker and artificial intelligence technologies to improve the training efficiency and safety. Machine learning algorithms could further personalize feedback, adapting to each individual’s biomechanics and physiological responses over time.

## 1. Introduction

CrossFit^®^ functional fitness programs have quickly become one of the fastest-growing training concepts, with over 15,000 affiliated centers and 5 million participants worldwide [1]. The combination of high-intensity physical benefits and a strong sense of community has driven CrossFit^®^ to unprecedented global popularity. A key factor influencing the adherence and continuation of practice may be psychological factors (like motivation), which encourage participants to engage consistently in exercise [2]. Individuals from diverse backgrounds, including those with obesity and seemingly healthy participants, are engaged in CrossFit^®^ primarily to enhance their health and exercise status and/or sports performance [2,3]. Like other high-intensity regimens, CrossFit^®^ enhances body composition, muscular strength, cardiorespiratory fitness and discrete health markers [3,4]. Beyond physical benefits, CrossFit^®^ practice can improve participants’ quality of life by influencing factors such as mental, social and functional well-being [2,5]. However, evidence increasingly suggests that the physiological demands of certain CrossFit^®^ workouts, combined with insufficient recovery, may heighten the risk of overload, fatigue and injury [3,6].

CrossFit^®^ workouts consist of high-intensity functional movements, typically combining gymnastics, metabolic conditioning and weightlifting exercises [7,8]. These routines are executed in a circuit format, with minimal rest, aiming for the fastest completion time or the highest number of repetitions over ~2 to 40 min [9,10]. Moreover, the workout of the day combines the development of strength and endurance in both the upper and lower body. By integrating strength exercises with workouts like Isabel, a resistance activity is created that demands both aerobic and anaerobic performance [6,9], recruiting different types of muscle fibers and fostering synergistic potential that can be adapted for hypertrophy goals [11]. In fact, it has been demonstrated that 12 weeks of regular CrossFit^®^ training can elicit a significant increase in maximal strength (~9 to 17%) and improvements in lean mass percentage (+1.05 kg). In addition, the CrossFit^®^ program has been scientifically shown to be effective in reducing body fat (−3.19 kg) [12].

CrossFit^®^ training aims to prepare participants for a wide range of workouts, emphasizing that constant variation is a core element of the methodology [1,8]. In competitions, the requirements for the workout of the day are announced to participants just a few minutes before the event, marking a key distinction from other sports, where athletes typically know in advance which discipline will be performed [8,13]. As in any sport, peak performance in CrossFit^®^ competitive events is achieved only after years of structured training and requires continuous progression that should be carefully monitored throughout the training process [8,13]. To evaluate fitness and monitor changes in work capacity, CrossFit^®^ incorporates benchmark workouts (Table 1), with Cindy and Fran being the most extensively assessed [7,14,15,16,17]. CrossFit^®^ workouts of the day adhere to standardized conditions, ensuring uniform performance comparisons among CrossFitters worldwide. These benchmarks vary in exercise composition, intensity, duration, exercise type and rest intervals [1,14]. Adjusting these variables influences the extent of fitness, performance gains and the risk of overload [1,8]. Some CrossFit^®^ workouts elicit substantial hormonal and metabolic disturbances, coupled with short-term heightened oxidative stress and inflammation [10,18,19].

The original CrossFit^®^ programming consisted of five weekly sessions (typically structured as 5 days of training followed by 2 days of rest, or 3 days of training with 1 day of rest), with most sessions featuring mixed content [20]. This approach, which combines high-intensity interval training methods with strength exercises, challenges the traditional principle of modality separation. However, it is crucial to account for adequate recovery time following high-intensity training, as insufficient recovery may negatively impact adaptive mechanisms [7]. Understanding the intricate relationship between energy availability and exercise-induced fatigue is essential in CrossFit^®^ workouts [14]. High training volumes and frequencies can elevate the risk of premature fatigue, increase the perceived exertion and reduce the movement quality [6,9]. In addition, CrossFitters often report pronounced fatigue, muscle soreness and a restricted range of motion in the 24–48 h post-workout [7,18]. The standardized loads in CrossFit^®^ substantially impact mobility, imposing added stress on the knee, hip and shoulder joints, which heightens the injury risk [6,19]. In addition, understanding the recovery timeline after sessions is crucial to minimize maladaptive responses from inadequate repair between training stimuli, informing effective exercise prescription and monitoring strategies [7,18].

Effective training and adaptation in sports and exercise require monitoring, quantifying and regulating both external and internal training loads [3,21]. However, its management poses a substantial challenge for researchers, coaches and CrossFitters. A substantial part of CrossFit^®^-related research focuses on identifying performance predictors through conventional laboratory tests [1,22]. However, given its unique nature, traditional testing protocols typically have limited applicability, since their physiological and biomechanical demands differ from those encountered in actual training environments [9,14]. Given that CrossFit^®^ exercises range from ~2 to 40 min, it becomes evident that CrossFitters rely on well-developed aerobic and anaerobic energy pathways [9,14]. Both these energy sources have traditionally been assessed in various laboratory-based cyclical sports using oxygen uptake (VO_2_) and blood lactate concentrations ([La^−^]), with the heart rate (HR) and rating of perceived exertion (RPE) serving as complementary indicators, especially in practical settings, where costly equipment and complex procedures pose challenges [23].

In the current work, we present physiological responses (using common physiological variables like VO₂, HR, blood pressure and [La^−^]) to different types of CrossFit^®^ workouts based on assessments conducted in real exercise environments. We also compare these responses with reference values established in the specialized literature for improvements in physical fitness. Concurrently, we also evaluate studies that have assessed changes in movement techniques, psychological well-being and recovery times, providing valuable insights for CrossFit^®^ researchers, coaches, practitioners and people in general that are interested in these specific routines.

## 2. Physiological Responses on CrossFit^®^

The varied stimuli in CrossFit^®^ workouts trigger a wide range of biological responses, resulting in a number of acute and chronic physiological adaptations observed in both experienced individuals and beginners [17]. Recent studies have demonstrated that the Fran workout performed at maximal effort, whether in an intermittent [14] or unbroken [7] format, induces high peak VO_2_ values (~46 to 49 mL∙kg^−1^∙min^−1^). Similarly, relevant peak VO_2_ values (~47 mL∙kg^−1^∙min^−1^) were observed in the Isabel workout (which was performed in ~120 s) [9], highlighting the importance of oxidative phosphorylation metabolism also in short-duration workouts. Studies that assessed the maximal and/or peak VO_2_ through standard treadmill tests in experienced CrossFitters reported values of 48 to 55 mL·kg^−1^·min^−1^, aligning closely with the interval displayed for the Fran and Isabel workouts [24,25]. Moreover, sustained HR responses exceeding 90% of its maximum value were observed across varied CrossFit^®^ workouts [26], nearing the levels typical of maximal-intensity exertion (as defined by the American College of Sports Medicine [27]). This exercise intensity may induce both acute and chronic adaptations, contributing to cardiovascular conditioning in CrossFitters (regardless of the session duration) [28].

An increase in HR is associated with a reduction in parasympathetic autonomic stimulation and a rise in sympathetic activity that, during exercise, is triggered by proprioceptors, the baroreceptor reflex and chemoreceptors [29]. Heart rate variability (HRV) is considered a non-invasive and time-efficient physiological marker for autonomic nervous system modulation and is easy to implement and cost-effective [30]. HRV assesses training load adaptations, aiding in control, fatigue detection and training adjustments. The root mean square successive difference in heart period series (RMSSD) curve indices indicate the internal load and reflect distinct responses between individuals [30,31]. A recent study demonstrated reductions in HRV and increases in sympathetic activity after performing the Fran (i.e., RMSSD: 49 vs. 76 ms), Megan (i.e., RMSSD: 49 vs. 72 ms) and Diane (i.e., RMSSD: 54 vs. 68 ms) workouts [31]. In intermittent exercises (like CrossFit^®^), autonomic modulation varies according to the intensity, implying that a low number of repetitions and moderate to high loads are key factors in cardiovascular stress [31]. Moreover, the additional time under tension resulting from the continuous execution of two or more exercises in a circuit mode, as typical in CrossFit^®^, has a more pronounced effect on the HRV response [31].

These repercussions for cardiorespiratory-related variables may impact the hemodynamic function of CrossFitters, increasing the hypotensive effect after the workout. In fact, studies have demonstrated a hypotensive effect following different CrossFit^®^ sessions, reducing the acute systolic and diastolic blood pressure, which may reduce underlying cardiovascular risk factors [31,32]. This hypotensive effect may be associated with a cardiac output that is not compensated for by a reduction in peripheral vascular resistance and an increase in sympathetic modulation [31]. In addition, a high volume of repetitions during a workout (like the 90 reps in the Fran routine) can potentiate motor unit recruitment, requiring greater sympathetic nervous system activity and the enhanced activation of mechanoreceptors and baroreceptors induced by mechanical blood flow occlusion [33].

CrossFit^®^ training sessions are often performed at near-maximal or maximal effort, resulting in a very significant metabolic stimulus [3,26]. The intensity of these sessions is usually quantified by assessing [La^−^], with values frequently exceeding 10 mmol·L^−1^ [9,26,28]. However, the increase in [La^−^] may vary depending on the specific CrossFit^®^ routine (like Cindy, Fran or Diane), with the workout volume, intensity and type of exercise as the primary influencing factors [28,34]. High [La^−^] levels are a key physiological indicator of anaerobic system functions (e.g., recruitment of type II muscle fibers, balance between aerobic and anaerobic metabolism and glycolytic capacity), indicating whether the exercises provide sufficient stimuli to induce adaptations [9,14]. This information is essential in determining the most effective training program for CrossFitters and give fundamental support for coaches to design an optimal training schedule (i.e., predominantly aerobic vs. anaerobic workouts).

Circuit training is widely recognized for its effectiveness in simultaneously improving muscular strength, power, endurance and aerobic and anaerobic fitness—key attributes in CrossFit^®^ [35]. This modality relies on the principles of concurrent training, strategically integrating activities aimed at enhancing endurance (e.g., rowing and running) and strength (e.g., weightlifting and bodyweight exercises) [35]. This systematic approach fosters the balanced development of multiple physical capacities during training sessions. One study showed that, after nine weeks of intervention with a CrossFit^®^-based program, untrained individuals had significant increases in maximal strength, with improvements in squat (10%) and shoulder press (4%) performance [36]. In addition, another study reported a ~14% increase in the weight lifted during the 5RM front squat after 16 weeks of CrossFit^®^-based exercise in recreationally active males and females [37].

Considering that strength gains directly contribute to the development of muscular power, and that CrossFit^®^ training programs include Olympic weightlifting exercises (e.g., snatch, clean and their variations) with high external loads and/or high repetition volumes [6], CrossFitters can expect improved power and muscular endurance (factors that can be crucial for muscle hypertrophy) [12]. In fact, following an intervention with a CrossFit^®^ session, substantial improvements in power, muscular endurance and flexibility were reported in both males and females [38,39]. Traditionally, the strength component is prioritized over endurance, as recommended in concurrent training [35,40]. However, the structure of a CrossFit^®^ session is flexible, and this order can be reversed with positive outcomes. The optimal sequence should be determined based on the primary goal, such as maximal strength, power or endurance, allowing workouts to be adjusted for more effective results [35].

It is widely recognized that high-intensity exercise induces acute changes in hormonal levels, including testosterone and cortisol, directly impacting protein synthesis, muscle regeneration and strength gains [41,42]. Testosterone, an anabolic hormone, is essential for protein synthesis, strength adaptation, muscle hypertrophy and psychological preparation for competitions, with transient elevations during exercise potentially increasing the maximal strength and training intensity [41,43]. In contrast, cortisol, a catabolic hormone, responds to stress by mobilizing energy through lipolysis and proteolysis, but elevated levels can inhibit testosterone’s anabolic effects [43]. An acute CrossFit^®^ session can induce marked fluctuations in hormonal profiles, with testosterone levels ranging from ~29 to 23 pg/mL and cortisol levels dropping from ~13 to 8 µg/dL after 48 h of recovery [44]. Over a chronic period of 6 months, male CrossFit^®^ practitioners demonstrated increased testosterone levels (~421 to 564 pg/mL) accompanied by reduced cortisol (~15 to 12 pg/mL) concentrations. Interestingly, this hormonal adaptation was not mirrored in female participants, whose levels remained unchanged [20].

The acute increase in testosterone can be explained by transient strength gains during training and repetitions with maximal effort, which generate additional overload, while the increase in cortisol is influenced by psychological factors, such as the perception of effort [41,45]. High-volume and high-intensity protocols, with short rest intervals between sets, induce more pronounced hormonal changes. This difference can be explained by the distinct neuromuscular, morphological and metabolic actions between the sexes [20,46]. While testosterone exerts its effects in men, estrogen plays a predominant role in women. These hormonal differences affect the muscle composition, with men having greater muscle mass and a higher number of type II fibers, resulting in higher testosterone levels [20,46].

## 3. Psychological Effects of CrossFit^®^

Recent studies emphasize the increasing focus on psychological factors in sports, investigating the health benefits of exercise and the mental dynamics that affect athletic performance [2,3,47]. The practice of CrossFit^®^ can improve both physical fitness indicators and participants’ mental health and cognitive performance [2,48,49]. Recent research shows that the motivation to engage in CrossFit^®^ is driven by intrinsic factors such as enjoyment, challenge and affiliation, along with a strong sense of community [50,51]. Self-determination theory explains that fulfilling the needs for autonomy, competence and relatedness supports long-term participation, while the scalability of exercises further enhances motivation [48]. In addition, social connection also plays a key role in sustaining engagement in the practice [2].

The communal and motivational nature of CrossFit^®^ is a crucial factor for long-term adherence and in maintaining a positive emotional state, making it an effective alternative to isolated or traditional training, which often lacks social interaction and mutual support [48,50]. The effects of CrossFit^®^ on mood are varied, with some studies indicating negative impacts due to its intensity, while others report improvements in mood after training [2]. The relationship between CrossFit^®^ and psychological health still requires further investigation, particularly in areas such as exercise dependence and body image [52,53]. While training has shown benefits for body image and well-being, few studies have examined its effects on self-esteem [54].

CrossFit^®^ can promote improvements in cognitive domains, such as short-term spatial learning, visual pattern separation and inhibitory control [49,55]. In addition, an acute session of CrossFit^®^ improved working memory in healthy individuals when compared to moderate-intensity aerobic exercise [56]. Although its focus is physical fitness, the physical stress induced during training may have positive effects on brain function by stimulating the production of neurotransmitters (like endorphins and dopamine), which are essential for the learning process and in maintaining motivation [57]. Studies also indicate that intense training can contribute to neuroplasticity, enhancing the brain’s ability to reorganize and form new neural connections, which facilitates adaptation to new challenges and situations [57].

## 4. Risks and Recovery Challenges

Given the high physiological stress induced by CrossFit^®^ workouts (even without fixed external loads), understanding the post-exercise recovery timeline is essential to optimize CrossFitters’ training [9]. In fact, CrossFit^®^ participants reported a higher RPE and more frequent intense training days per week than what is recommended by the American College of Sports Medicine [58]. Recent cross-sectional studies assessing recovery in experienced CrossFitters using plasma biomarkers (e.g., creatine kinase concentrations) and performance-related tests (e.g., countermovement jump) indicate that individuals often do not fully return to baseline values even 24 h post-workout [7,14,18]. After two consecutive days of competition in CrossFit, a participant showed creatine kinase values of 77,590 U/L three days after the competition [59], accompanied by myalgia and abnormal liver function tests, with levels remaining elevated until the 10th (3034 U/L) and the 25th day (1257 U/L). Creatine kinase concentrations can rise due to exercise-induced muscle damage from intense and prolonged training, remaining elevated for up to 96 h after predominantly eccentric exercises, which extends the required recovery time before engaging in the next training session [60]. In addtion, the significant reduction in countermovement jump ability (i.e., jump height: 8%, peak force: 6% and maximum velocity: 4%) was associated with decreased neuromuscular function [7], particularly affecting type II muscle fibers, which are essential for high-load external resistance training [7]. Coaches and CrossFitters can use this information to better prepare the following exercise session, focusing on different body parts or modifying the intensity to manage their recovery and training load [21].

The association between fatigue and biodynamic movements is especially important in the context of CrossFit^®^ workouts. Olympic weightlifting exercises are an integral part of CrossFit^®^ programming and competing, used in both single-movement focused training sessions (e.g., snatches in the Isabel workout) and metabolic conditioning routines [6,32]. Recent research identified changes in velocity and power production during the Isabel routine, highlighting the impact of high-intensity training on the individual power profile. Despite declines in biomechanical factors (like hip flexion), CrossFitters applied adaptive pacing techniques to maintain high performance levels despite increasing fatigue [6]. With a standardized load (61 kg) and fixed repetitions (30 times) in the Isabel workout, CrossFitters adjust the tempo of contractions to moderate fatigue, reflecting dynamic adjustments in movement mechanics. The primary cause of angular changes appears to be lower limb fatigue, which reduces the bar lift velocity and power during the final phases [6].

## 5. Future Directions

Future studies on CrossFit^®^ should investigate the long-term cardiovascular health effects of repeated workouts, especially among recreational participants and across different levels of training experience. While acute reductions in systolic and diastolic blood pressure have been observed following CrossFit^®^ sessions, it remains unclear whether these hypotensive effects persist with long-term participation. In addition, the impact of repeated exposure to high-intensity CrossFit^®^ on the VO_2_ max, HRV, sympathetic and parasympathetic balance and overall cardiovascular health warrants investigation for multiple reasons, particularly in optimizing training protocols to improve cardiovascular function and aerobic capacity (supporting sustained health benefits). Understanding how different CrossFit^®^ workout types (e.g., metabolic conditioning and strength training) affect VO_2_ and autonomic regulation will provide insights for the design of physical fitness programs that enhance heart health while minimizing cardiovascular risks.

Furthermore, upcoming research could examine sex-based differences in cardiovascular adaptations, as hormonal influences and the muscle composition may contribute to the distinct responses and recovery requirements in males and females. Recovery strategies after intense CrossFit^®^ sessions also need further investigation to promote both muscular and cardiovascular recovery. Although high-intensity workouts impose considerable metabolic stress and elevate the VO_2_ levels, there is limited knowledge of how recreational CrossFitters recover from such exertions. Studies should analyze the recovery timeline using biomarkers (e.g., creatine kinase, total antioxidant status and malondialdehyde) and functional performance tests (e.g., countermovement jumps and prone planks) to develop effective strategies that support muscle function and cardiovascular recovery. These insights could aid in injury prevention, promote optimal recovery and support long-term physical activity (ultimately contributing to overall health).

Integrating advanced technologies like surface electromyography and artificial intelligence for real-time physiological and biomechanical data analysis (e.g., muscle activation patterns, kinematics and kinetics) will enable wearable devices to provide immediate feedback, optimizing performance and movement quality. By capturing joint angles, limb velocities and muscle forces, these devices could help CrossFitters to correct inefficient movement patterns, reducing the injury risk and enhancing the training effectiveness. Up-to-date technologies have the potential to democratize sophisticated data analytics, bringing insights previously limited to research and high-performance settings to all CrossFit^®^ practitioners. Machine learning algorithms could further personalize feedback, adapting the advice to each individual’s biomechanics and physiological responses over time. This approach could improve the training safety and effectiveness by offering real-time customized guidance aligned with each participant’s biomechanics and conditioning level.

## 6. Practical Applications

CrossFit^®^ workouts have a wide variety of practical applications in enhancing physical fitness through high-intensity exercises that stimulate both aerobic and anaerobic adaptations. Studies show that performing these exercises at maximum effort can elevate the peak VO_2_ and HR close to maximal levels, strengthening the cardiovascular capacity and oxidative metabolism. Sustained HR responses align with typical endurance training, fostering cardiovascular health. The high-repetition nature of these workouts aids in muscle recruitment, increasing motor unit activation and enhancing neuromuscular coordination. CrossFit^®^ sessions also generate a hypotensive effect, with post-exercise blood pressure reductions that may mitigate cardiovascular risks. The metabolic demands of these workouts, often indicated by high [La^−^], effectively challenge anaerobic systems, promoting muscular endurance. The combination of strength and endurance exercises promotes balanced progress in various physical abilities, making it essential to structure CrossFit^®^ sessions according to the primary goal(s). In contrast, consistent participation necessitates strategic recovery planning due to prolonged fatigue and muscle damage, guiding CrossFitters and coaches in optimizing the training intensity and scheduling.

## 7. Conclusions

CrossFit^®^ workouts are effective in enhancing physical fitness, as evidenced by their superior cardiorespiratory, metabolic, maximal strength and muscular endurance responses compared to moderate-intensity exercises. CrossFit^®^ not only improves physical fitness but also enhances mental health and cognitive performance through its communal motivating environment. However, the substantial increases in biochemical marker concentrations, metabolic stress and perceived exertion, along with the decrease in the countermovement jump test post-workout performance, underscore the importance of proper recovery between sessions. These findings emphasize the need to carefully manage training loads and recovery strategies to optimize performance, prevent overtraining and ensure sustained improvements in fitness and overall health.

## Figures and Tables

**Table 1:** A comprehensive overview of the exercises and structural elements inherent in CrossFit^®^ benchmark workouts.

CrossFit^®^ Benchmark Workouts Known as the Girls			
Exclusively bodyweight movements	**Cindy** As many rounds and repetitions as possible (for 20 min) Five pull-ups 10 push-ups 15 air squats	**Chelsea** One full round every min (for 30 min) Five pull-ups 10 push-ups 15 air squats	**Barbara** 5 rounds (for time) 20 pull-ups 30 push-ups 40 sit-ups 50 air squats
Incorporating bodyweight movements plus wall balls and/or kettlebell	**Kelly** Five rounds (for time) 400-m run 30 box jumps (24/20) 30 wall ball shots (20/14 lb)	**Helen** Three rounds (for Time) 400-m run 21 kettlebell swings 12 pull-ups	**Eva** Five rounds (for time) 800-m run 30 kettlebell swings 30 pull-ups
Incorporating a combination of barbell plus calisthenics movements	**Fran** 21-15-9 (repetitions for time) Thrusters (95/65 lb) Pull-ups	**Jackie** (for time) 1000-m row 50 thrusters (45/35 lb) 30 pull-ups	**Amanda** 9-7-5 (repetitions for time) Muscle-ups Squat snatches (135/95 lb)
Intensive barbell workouts with heavy weights	**Isabel** (for time) 30 snatches (135/95 lb)	**Grace** (for time) 30 clean and jerks (135/95 lb)	**Linda** 10-9-8-7-6-5-4-3-2-1 (repetitions for time) Deadlift (1½ bodyweight) Bench press (bodyweight) Clean (¾ bodyweight)

## Data Availability

No new data were created or analyzed in this study.
